# Self-Powered Intelligent Water Droplet Monitoring Sensor Based on Solid–Liquid Triboelectric Nanogenerator

**DOI:** 10.3390/s24061761

**Published:** 2024-03-08

**Authors:** Lijie Zhu, Likang Guo, Zhi Ding, Zhengqian Zhao, Chaoran Liu, Lufeng Che

**Affiliations:** 1 College of Information Science and Electronic Engineering, Zhejiang University, Hangzhou 310027, China; zljsherry@zju.edu.cn (L.Z.); 22160565@zju.edu.cn (Z.D.); zhaozhengqian@zju.edu.cn (Z.Z.); 2 Center for Microelectronics, Shaoxing Institute, Zhejiang University, Shaoxing 312035, China; 3 Ministry of Education Engineering Research Center of Smart Microsensors and Microsystems, College of Electronics and Information, Hangzhou Dianzi University, Hangzhou 310018, China; glk0906@163.com; 4 State Key Laboratory of Fluid Power and Mechatronic Systems, Zhejiang University, Hangzhou 310058, China

**Keywords:** self-powered sensor, water droplet monitoring, solid–liquid triboelectric nanogenerator, intelligent analysis, automobile automatic wiper

## Abstract

Real-time monitoring of rainwater is a critical issue in the development of autonomous vehicles and smart homes, while the corresponding sensors play a pivotal role in ensuring their sensitivity. Here, we study a self-powered intelligent water droplet monitoring sensor based on a solid–liquid triboelectric nanogenerator (SL-TENG). The sensor comprises a SL-TENG, a signal acquisition module, a central processing unit (CPU), and a wireless transmission module, facilitating the real-time monitoring of water droplet signals. It is worth noting that the SL-TENG has self-powering characteristics and can convert the kinetic energy of water droplets into electrical energy. The excellent output performance, with open-circuit voltage of 9 V and short-circuit current of 2 μA without any treatment of the SL-TENG, can provide an effective solution to the problem that traditional sensor need battery replacement. In addition, the SL-TENG can generate stable amplitude electrical signals through water droplets, exemplified by the absence of decay in a short-circuit current within 7 days. More importantly, the sensor is equipped with intelligent analytical capabilities, allowing it to assess rainfall based on variables such as amplitude and frequency. Due to its excellent stability and intelligent analysis, this sensor can be used for roof rainwater monitoring, intravenous administration monitoring, and especially in automobile automatic wipers and other fields.

## 1. Introduction

Since Professor Wang invented the triboelectric nanogenerator (TENGs) in 2012 [1], this technology has achieved rapid development. The principle of a solid–liquid triboelectric nanogenerator is different from that of a solid–solid triboelectric nanogenerator. It transforms the kinetic energy of water into electrical energy, suitable for applications in both energy storage [2,3,4,5,6] and signal detection [7,8,9]. In 2014, Wang et al. realized energy harvesting from rainwater for the first time, but the output power was only 0.02 W/m^2^ [10]. However, the traditional TENGs universally employed interfacial effect for output, resulting in severe limitations on output efficiency [11]. In 2019, Wang et al. transformed the conventional interfacial effect into a bulk effect [12], significantly enhancing the overall output performance of SL-TENG. Zhang et al. reported a universal single electrode droplet-based electricity generator (SE-DEG) that allows for converting water droplet kinetic energy into electric energy on any surfaces of artificial or natural materials [13]. Dong et al. achieved direct current (DC) output by integrating the rectification, energy conversion, and storage components into the same device [14]. In addition, Xu et al. analyzed the relationship between the time-domain current waveform and the movement of the droplet [15].

As our understanding of TENGs advances, an increasing number of sensors are being developed [16,17,18,19]. Cui et al. studied tube-based TENG for self-powered blockage detecting and air pressure monitoring [20]. Shen et al. studied a wearable sweat sensor that can analyze the abundant composition of solutes and metabolites in sweat to reflect the health state of the wearers in real time [21]. Liu et al. fabricated a highly sensitive and self-powered acid rain sensor based on a TENG using a doping method [22]. A large amount of research focuses on TENG materials [23,24,25], structures [26,27,28,29,30], and other aspects to improve the output performance. However, there is a relatively limited body of studies exploring the application of TENGs in self-powered water droplet monitoring.

In everyday scenarios, water droplet monitoring sensors are commonly employed for automobile automatic wipers, wireless alarm systems, and intravenous administration monitoring. The operational principle of these sensors involves positioning an infrared-emitting probe at the location where water droplets are to be monitored and placing an infrared receiver where an external power supply is needed [31]. Currently, the majority of water droplet monitoring sensors rely on battery power, exhibiting drawbacks such as poor sensitivity, high cost, weak anti-interference ability, and significant environmental impact. Furthermore, these sensors often lack a comprehensive system analysis [32,33,34,35,36,37], which would integrate functions such as signal collection, processing, transmission, display, and analysis. A comprehensive system would enable real-time observation of rainfall, facilitating precise control of wiper speed. Hence, it is highly valuable to develop a water droplet monitoring sensor with elevated sensitivity and self-power supply capability. Due to the electrostatic induction and frictional electricity principles inherent in TENGs, self-powered sensors based on TENGs can use electrical signals generated by water droplets to power themselves. Although there are already self-powered sensors [38], there is no representation of self-powered water droplet sensors.

In this study, we explore a water droplet monitoring sensor built upon a SL-TENG. The SL-TENG is composed of PTFE film and Cu electrode, which not only has a simple structure but also a stable output performance. Each drop of water can produce a steady amplitude electrical signal, and the short-circuit current does not decay for 7 days. In addition, the SL-TENG has a good self-powering performance and can convert the kinetic energy of water into electric energy. The hydrophobic treatment of copper electrode with the perfluoro compound reduces the noise of an electrical signal from 0.23 μA to 0.13 μA, which lays a good foundation for signal acquisition. The above characteristics of the SL-TENG are conducive to the real-time monitoring of rainfall. Crucially, this sensor can make judgments on rainfall based on multiple variables such as frequency and amplitude, thus accurately controlling the speed of a wiper. When light rain is detected, the wiper does not move. When middle rain is detected, the wiper moves slowly. When heavy rain is detected, the wiper moves quickly. Additionally, the signal is transmitted to the host computer platform through the circuit system acquisition module, signal processing module, and Bluetooth wireless transmission module, enabling real-time remote monitoring and a wireless alarm. This research provides new directions for areas such as automatic driving and wireless alarms on rainy days. Although this is just a small attempt, it provides new ideas for the development of self-powered intelligent sensors.

## 2. Materials and Methods

### 2.1. Fabrication of SL-TENG_S_

The SL-TENG is composed of PTFE film and Cu electrode. The PTFE film comes in thicknesses of 30 μm, 50 μm, 80 μm, and 100 μm, with each film measuring 4 cm × 4 cm. On the one hand, a copper film with a size of 4 cm × 4 cm is cut from the copper foil tape and combined with a PTFE film as the lower electrode. Simultaneously, the perfluoro compound is applied to the copper foil tape and cured at 70 °C in an oven for 30 min to enhance its hydrophobic properties [39]. Next, we cut a piece of 4 cm × 1.2 cm copper tape that has been treated for hydrophobicity and adhere it onto the PTFE film as the upper electrode. Finally, we stick the device on PMMA. In this experiment, the FEP and Kapton film used were both 100 μm.

### 2.2. Establishment of Sensors Based on the SL-TENG

First, the impedance matching is achieved through the voltage-following module. Then, the overall voltage is promoted by 1.5 V through the TLC074CN operational amplifier and resistor module to comply with the sampling range of the STM32. Finally, the STM32 collects the electrical signal and transmits it to the host computer platform through Bluetooth to achieve the real-time display of signals.

### 2.3. Measurements

Measurements are conducted using the Keithley 6514 Electrometer (Tektronix, Shanghai, China) to precisely monitor open-circuit voltage, short-circuit current, and charge transfer. The droplet volume is adjustable by flexibly manipulating the needle, while the syringe pump is coordinated to alter variables such as frequency. Simultaneously, through the host computer platform constructed with Qt Creator 5.12.1 software, users can observe real-time waveform changes and receive alarm information. This ensures the attainment of a comprehensive real-time data display and monitoring functions throughout the experiment.

## 3. Results and Discussion

### 3.1. Working Principle of the SL-TENG

**Figure 1:**
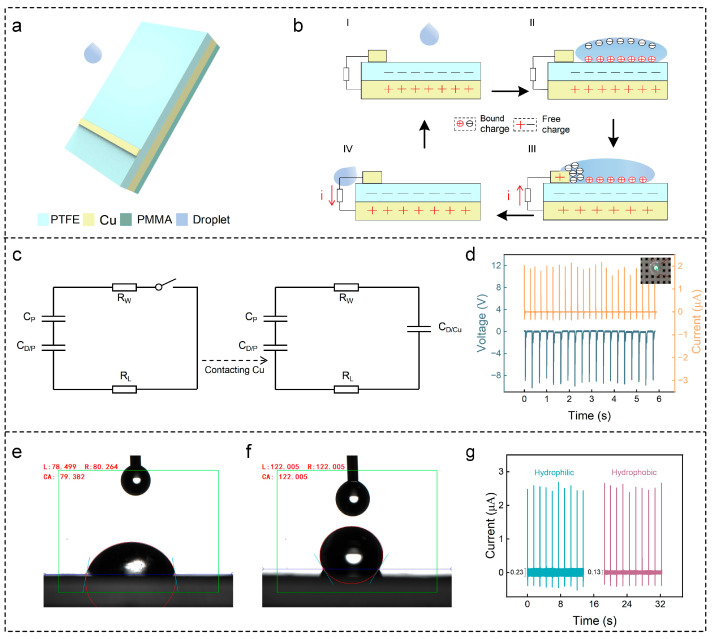
(**a**) Structure of the SL-TENG; (**b**) schematic diagram of the SL-TENG; (**c**) circuit model of the SL-TENG; (**d**) basic output performance of open-circuit voltage and short-circuit current based on SL-TENG; (**e**) hydrophobic angle of upper copper electrode before hydrophobic treatment; (**f**) hydrophobic angle of upper copper electrode after hydrophobic treatment; (**g**) comparison of short-circuit current before and after hydrophobic treatment of the SL-TENG.

Based on the flow characteristics of water droplets, we developed an intelligent water droplet monitoring sensor implemented with an SL-TENG. The SL-TENG is composed of a PTFE film, two Cu electrodes, and a piece of PMMA (Figure 1a). Unlike traditional SL-TENG that relies on an interfacial effect for output and exhibits a limited output performance, our SL-TENG achieves a terrific output power of 3.2 μW without the need for additional processing. The working mechanism of the SL-TENG relies on the friction between water droplets and insulating materials, and the insulating materials need to be hydrophobic. Consequently, we designed a water droplet monitoring sensor characterized with a simple structure, good stability, and an optimized size. Its minimum open-circuit voltage is 4 V and its minimum short-circuit current is 0.8 μA, which is suitable for the range allowed by the circuit.

The SL-TENG operates primarily in four distinct states: the initial state, water droplet dripping, water droplet diffusion, and water droplet sliding (Figure 1b). In the initial state, the charge does not flow. As water droplets fall and fail to contact the upper electrode, the water droplets spread on the PTFE film, and the water droplets and the PTFE film accumulate positive and negative charges, respectively. The positive charge on the water droplets and the negative charge on the PTFE film interact with each other, leading to a no-charge-transfer process. When water droplets continue to spread and make contact with the upper electrode, the circuit is turned on. The accumulated charge is instantly released, with the positive charge on the lower electrode transferred to the upper electrode through the peripheral circuit. As shown in Figure 1c, where *C_P_*, *C_D/P_*, and *C_D/Cu_* represent the capacitance formed by water–PTFE and PTFE–Cu, water and PTFE, and water and Cu, respectively. *R_W_* and *R_L_* are the resistances associated with water and the load, respectively. As water droplets slide off the upper electrode, the positive charge on the electrode returns to the lower electrode. The changing contact areas between water droplets and the upper electrode, as well as between water droplets and the PTFE film, result in a relatively small reverse current. In summary, continuous water droplets can generate periodic electrical signals. For a more comprehensive understanding of the SL-TENG’s functionality, we analyze *Q_max_* as a function of the Weber number. This number is defined as We = *ρDv^2^/γ*, where *D*, *v*, and *γ* are the diameter, impact velocity and surface tension of the droplet, respectively [12]. The charge transfer trend aligns with that of the Weber number. Additionally, the open-circuit voltage *V* is expressed in Equation (1) as follows, where *d_PTFE_* and *ε_P_* denote the thickness and dielectric constant of the PTFE film, respectively, and *Q_max_* and *S_PTFE_* represent the transferred charge and the contact area between the water droplet and the PTFE film, respectively:

(1) $$V=Q_{max}d_{PTFE}/\varepsilon_{P}S_{PTFE}$$

The basic output signal of the SL-TENG is shown in Figure 1d. When the water droplets are not falling, there is no output signal. However, when water droplets fall, the open-circuit voltage is about 8 V and the short-circuit current is about 2 μA. Moreover, the SL-TENG can light up the led lamp without any external power supply, and each water droplet can generate an electrical signal. This demonstrates the ability of the SL-TENG to harvest the water droplet energy to power low-energy electronic devices. Given that the water contact angle of the Cu electrode above the original TENG sensor is less than 90 °C (Figure 1e), water droplets lingering on the electrode can adversely impact the subsequent water droplet’s output performance. To address this, the perfluoro compound was applied to increase hydrophobic properties. As shown in Figure 1f, after hydrophobic treatment, the hydrophobic angle of the upper electrode reached 122.005 °C, showing excellent hydrophobic performance. Subsequently, we compared the short-circuit current before and after hydrophobic treatment of the device, with the results presented in Figure 1g. The SL-TENG exhibited a substantial reduction in output noise, decreasing from 0.23 μA to 0.13 μA after the hydrophobic treatment.

### 3.2. Investigation on the Output Law of the SL-TENG

**Figure 2:**
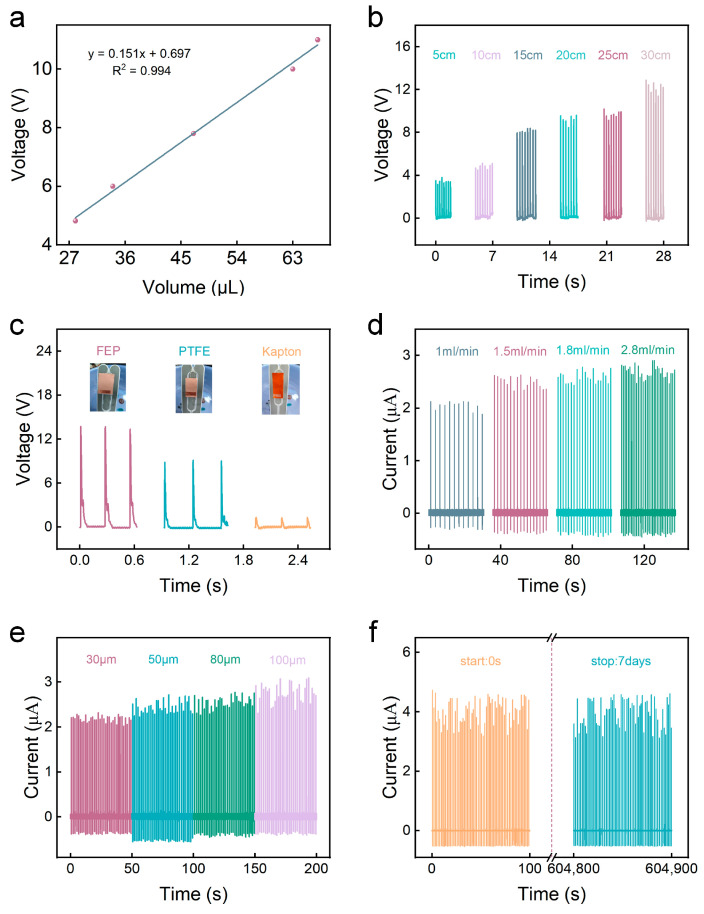
(**a**) The open-circuit voltage of the SL-TENG under different water droplet volumes; (**b**) the open-circuit voltage of the SL-TENG at different drop heights; (**c**) the open-circuit voltage of the devices are based on FEP film, PTFE film and Kapton film, respectively; (**d**) the short-circuit current of the SL-TENG at different frequencies; (**e**) the short-circuit current of devices with different film thicknesses; (**f**) stability test results of the SL-TENG.

In order to optimize the output performance and test sensor sensitivity, some structural parameters (Figure 2) were systematically investigated, including water droplet volume, drop height, falling frequency, film material, film thickness and device stability, etc. In order to control variables, the SL-TENGs used in the tests were all composed of a PTFE film and two Cu electrodes, unless otherwise specified. Obviously, as the volume of the water droplets increase, the peak value of open-circuit voltage continues to increase (Figure 2a). It shows that the SL-TENG produces a sensitivity of 0.151. This phenomenon is primarily attributed to the increase in droplet diameter, consequently augmenting the Weber coefficient and impacting the open-circuit voltage. The drop height of water droplets also has a significant impact on the charge accumulation and effective friction area. The higher the drop height, the larger the maximum spreading area of the water droplets, resulting in a greater amount of charge accumulation. Additionally, following the principles of the Weber function, an escalation in impact velocity corresponds to a higher accumulated charge. Consequently, when a loop is formed, the open-circuit voltage is elevated. As depicted in Figure 2b, when the drop height increases from 5 cm to 30 cm, the open-circuit voltage increases from 4 V to 12 V.

To assess the impact of various film materials on water droplets output performance, we selected three materials—FEP, PTFE, and Kapton—for output tests under fixed conditions of drop height (15 cm), water droplet volume (63 μL), and frequency (0.170 mL/s) (Figure 2c). It can be seen from the figure that the open-circuit voltage of FEP can reach up to 14 V, while the open-circuit voltage of Kapton is only 2 V. The open-circuit voltage of PTFE film is 9 V. After consulting the pertinent information, FEP possesses the highest electronegativity, while Kapton has the weakest electronegativity, aligning with our experimental results. Furthermore, we explored the output of water droplets at different frequencies. As illustrated in Figure 2d, with the water droplet falling frequency increasing from 1 mL/min to 2.8 mL/min, the short-circuit current escalates from 2.2 μA to 3.1 μA. This phenomenon may be attributed to the increased falling frequency leading to reduced surface tension. Therefore, the amount of accumulated charge increases, and the output performance improves.

Referring to Equation (1), it becomes evident that the device’s open-circuit voltage is directly proportional to the film’s thickness. To validate the correlation between SL-TENG output performance and film thickness, we conducted tests on short-circuit current at film thicknesses of 30 μm, 50 μm, 80 μm, and 100 μm. The results in Figure 2e distinctly illustrate that as the film thickness increases, the short-circuit current output correspondingly rises, aligning seamlessly with the anticipated outcomes. Subsequently, we performed a working stability test on the SL-TENG, testing a set of data every seven days to confirm its durability. As shown in Figure 2f, the amplitude of the short circuit current remains stable for 100 s and does not decay after 7 days.

### 3.3. Rainfall Test Characteristics and Signal Acquisition System of Water Droplet Monitoring Sensor

#### 3.3.1. Output Performance of the SL-TENG under Different Rainfall

To evaluate the output performance of the SL-TENG across varying rainfall intensities, we used a syringe pump and industrial distilled water to replicate rainwater descent conditions. Upon reviewing the relevant information, we found differences in the droplet volume, drop height, and falling frequency of water droplets corresponding to distinct rainfall intensities [40]. Moreover, it is evident from Figure 2 that this TENG exhibits the highest sensitivity to water droplet volume and drop frequency. Consequently, this study primarily introduces different water droplet volumes and drop frequencies to emulate diverse rainwater descent scenarios. In natural settings, the diameter of raindrops in light rain typically measures less than 2 mm, with middle rain featuring diameters ranging between 2 mm and 4 mm, and heavy rain featuring diameters generally exceeding 4 mm. Considering these natural phenomena, we selected a droplet diameter of 2 mm with a falling interval of 1.6 s to simulate light rain, a droplet diameter of 3.5 mm with a falling interval of 1 s to simulate middle rain, and a droplet diameter of 5 mm with a falling interval of 0.6 s to simulate heavy rain.

**Figure 3:**
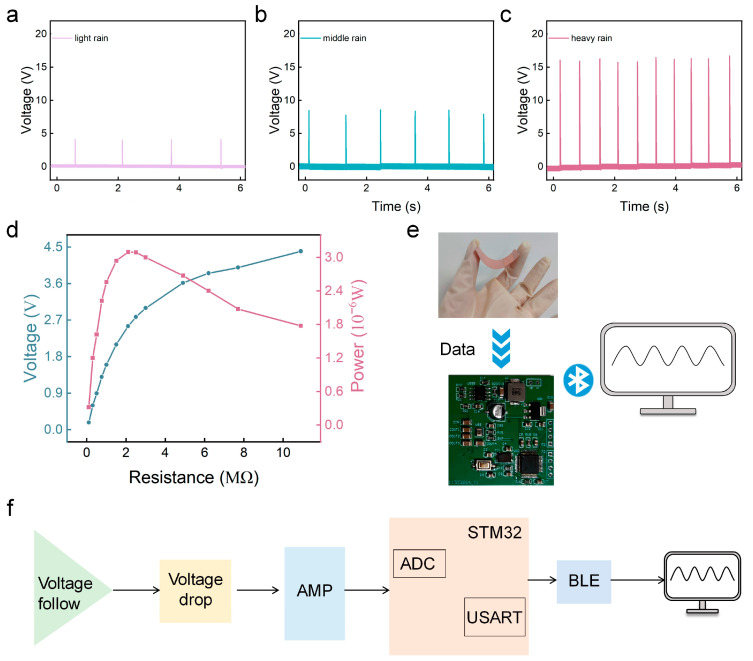
(**a**–**c**) Output performance of the SL-TENG under light rain, middle rain and heavy rain; (**d**) the open-circuit voltage and power of the water droplet monitoring sensor change as the external resistance changes; (**e**) flow chart of the workflow of the water droplet monitoring sensor; (**f**) schematic diagram of water droplet monitoring sensor.

The simulated rainfall output results are presented in Figure 3a–c, where noticeable variations in open-circuit voltage and frequency are apparent across the different rainfall intensities. Specifically, the open-circuit voltage for light rain registers at a mere 4 V, whereas heavy rain yields an impressive open-circuit voltage of up to 16 V. This disparity is further substantiated by the data presented in Table 1, where *Q_si_* and *V_se_* are the transferred charge and voltage integral, respectively. Through the analysis of the peak value of open-circuit voltage, frequency, charge amount, and voltage integral, the water droplet monitoring sensor can distinguish light rain, middle rain, and heavy rain.

#### 3.3.2. The Wireless Processing System of the Water Droplet Monitoring Sensor

The miniaturization, portability, and integration requirements for intelligent water droplet monitoring sensors necessitate a signal-processing system with a small size and intelligent processing capabilities. Therefore, we devised a microcircuit board, measuring just 4 cm × 4 cm. The comprehensive structure of the entire water droplet monitoring sensor is depicted in Figure 3e. The electrical signal generated by the SL-TENG was collected and processed through the circuit board, and then transmitted to the display screen through wireless transmission to display the monitoring results in real time. The detailed functioning of the microcircuit board is elucidated in Figure 3f, encompassing the collection and processing of electrical signals and the wireless transmission module. Given the high internal resistance of the SL-TENG (Figure 3d), we adopted voltage following negative feedback to reduce the input impedance from the MΩ level to the KΩ level, aligning with the analog-to-digital conversion range. Moreover, as the output voltage of the water droplet monitoring sensor does not fall within the sampling range of the microcontroller (STM32F103C8T6, STMicroelectronics, Geneva, Switzerland), we integrated the TLC074CN operational amplifier and operational amplifier circuit to increase the overall voltage by 1.5 V. The CPU conducted targeted digital filtering on the sampled signal, transmitting the data to the host computer platform via Bluetooth for real-time display.

### 3.4. System Debugging and Complex Environment Testing of the Water Droplet Monitoring Sensor

**Figure 4:**
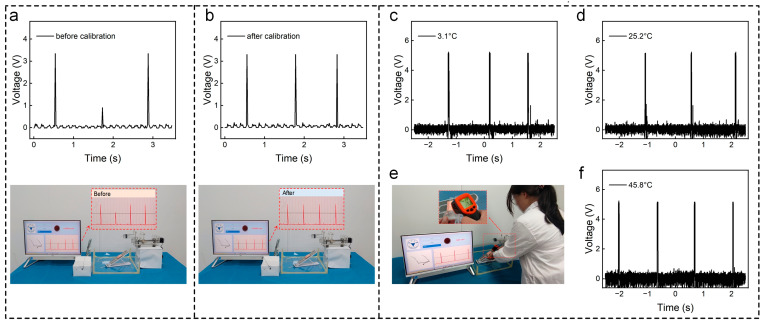
(**a**) Output of water droplet monitoring sensor before calibration; (**b**) output of water droplet monitoring sensor after calibration; (**c**) the open-circuit voltage of the SL-TENG at 3.1 °C; (**d**) the open-circuit voltage of the SL-TENG at 25.2 °C; (**e**) testing of the water droplet monitoring sensor at different temperatures; (**f**) the open-circuit voltage of the SL-TENG at 45.8 °C.

Due to the volume effect inherent in the SL-TENG, it exhibits a high output signal-to-noise ratio with relatively minimal noise impact. However, given the elevated output frequency of the SL-TENG and the need to adapt to more complex environments, we fine-tuned the baud rate to 38,400. This adjustment aims to ensure the transmission of valid data and prevent data transmission failures at peak points. We used the sscom serial debugger to collect the data transmitted via Bluetooth. The experimental results demonstrate that this method significantly enhances the output signal (Figure 4a,b). Moreover, through the human–computer interaction system, we successfully mitigated misjudgments and false alarms on the host computer platform. In order to explore the stability of the SL-TENG at different temperatures, a heat gun and ice cubes were used as temperature sources (Figure 4e). We used ice cubes to lower the SL-TENG’s temperature to 3.1 °C, and a heat gun to raise the SL-TENG’s temperature to 25.2 °C and 45.8 °C. Subsequently, we used an oscilloscope to collect the output signals of the SL-TENG device at different temperatures. The experimental results show that the noise of the open-circuit voltage of SL-TENG will increase at a high temperature, but it has no impact on the performance of the device (Figure 4c,d,f). Leveraging these characteristics, we have established a highly stable, intelligent water droplet monitoring sensor.

### 3.5. Assisted Rainwater Monitoring

By configuring a highly sensitive and stable sensor, we successfully built a self-powered water droplet monitoring sensor that can monitor rainfall in real time, including light rain, middle rain, and heavy rain. Compared with the traditional rainwater detector, the water droplet monitoring sensor based on SL-TENG can compare multiple parameters, such as amplitude and frequency. The sensor has the characteristics of portability, high sensitivity, stability, and embeddability.

**Figure 5:**
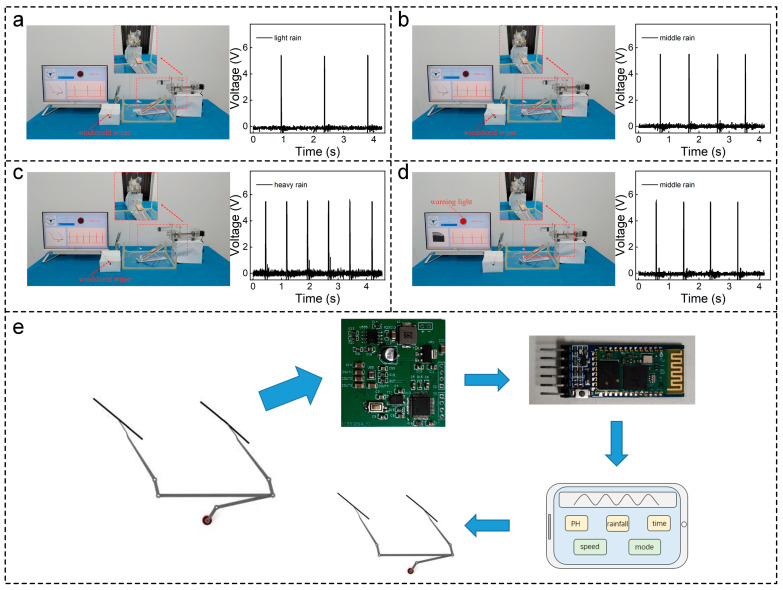
(**a**–**c**) Water droplet monitoring sensor identifies light rain, middle rain, and heavy rain; (**d**) the water droplet monitoring sensor detects that the car window is not closed on rainy days; (**e**) overall diagram of car automatic wiper.

Due to the constant changes in cloud cover, individuals may encounter diverse weather conditions throughout their journeys, ranging from heavy rain and light rain to sunny days. Such changing weather conditions can distract drivers from concentrating on the road. Considering the open-circuit voltage of SL-TENG exceeding the sampling range of the CPU, this paper distinguishes the rainfall by frequency. By accurately identifying the frequency of various rain scenarios, such as light rain, middle rain, and heavy rain (Figure 5a–c), our sensor provides precise guidance for the wiper system. This effectively reduces the probability of traffic accidents. When the water droplet monitoring sensor detects light rain, the wiper simulator remains inactive; for middle rain, the wiper simulator rotates at a low speed; and for heavy rain, the wiper simulator rotates rapidly. For a detailed demonstration of these operational effects, please refer to Supplementary Materials Video S1.

Furthermore, the sensor can promptly issue an alarm upon detecting rainwater. During outdoor breaks, car owners might face challenges in swiftly responding to weather changes and closing their car windows in rainy weather. When the water droplet intelligent monitoring sensor identifies rain signals and detects that the car window remains open, it triggers an alarm signal to alert the car owner to promptly close the window (Figure 5d). The corresponding video is available in Supplementary Materials Video S2. Additionally, the water droplet monitoring sensor can be integrated with the window-close sensor to automatically close the windows on rainy days.

Research shows that rain on windshields degrades the object detection performance of deep learning-based vision detectors. In autonomous driving, automatic car wipers play an important role in determining obstacles on the roadway conveniently and effectively. With the continuous development of artificial intelligence, the intelligent water droplet monitoring sensor is expected to be closely integrated with the car display screen to achieve precise control of wiper speed (Figure 5e). This integration presents a novel approach to autonomous driving on rainy days, contributing to automotive technology innovation and the anticipated enhancement of people’s travel experiences. We eagerly anticipate witnessing further innovations akin to this, which promise to enhance both the portability and safety aspects of the automotive industry.

## 4. Conclusions

In summary, we have successfully developed a self-powered intelligent water droplet monitoring sensor based on SL-TENG. This sensor has been successfully applied to automobile automatic wipers and wireless alarm systems. The Cu electrode was coated with the perfluoro compound to impart excellent hydrophobicity. We conducted tests on droplet volume, drop height, falling frequency, film material, film thickness, and device stability to investigate their impact on the SL-TENG output performance. Due to its self-powered characteristics, the device can power a led lamp without any additional processing. Furthermore, in terms of rainwater detection, the sensor has demonstrated outstanding performance. With a high output signal-to-noise ratio, it is capable of extracting multiple parameters, significantly enhancing the accurate identification of rainfall. The water droplet monitoring sensor possesses advantages such as self-powering, miniaturization, and intelligence. Although this is just an initial attempt, this comprehensive technological integration can pave the way for a new direction in self-powered intelligent-sensor development.

## Figures and Tables

**Table 1:** Core parameters of different rainfall levels obtained from SL-TENG.

Different State	Frequency (Hz)	Amplitude (V)	*Q_si_* (nC)	*V_se_* (mL)
Light rain	0.62	4.14	1.5	25.0821
Middle rain	1.00	8.37	3.5	61.5686
Heavy rain	1.67	16.62	5.0	201.9780

## Data Availability

Data are contained within the article.

## Supplementary Materials

The following supporting information can be downloaded at: https://www.mdpi.com/article/10.3390/s24061761/s1, Video S1: Simulate the working status of automatic car wipers under different rainfall amounts. Video S2: An alarm occurs if the window is not closed on a rainy day.
